# The Use of Neodymium-Doped Yttrium Aluminum Garnet Laser for Treatment of Hypertrophic Port-Wine Stain

**DOI:** 10.1155/2022/8788417

**Published:** 2022-01-25

**Authors:** Brittany Miles, Andrew Armenta, James Mackey

**Affiliations:** ^1^University of Texas Medical Branch, Galveston, Texas, USA; ^2^Deja You Medical Aesthetics, League City, Texas, USA

## Abstract

Port-wine stains (also called nevus flammeus) are congenital malformations of the capillaries and postcapillary venules. They occur in 0.1–2% of newborns without sex predilection. Although PWS lesions are flat early in life, with age, they become hypertrophic and darker. Pulsed dye laser therapy is the standard of care for treating these lesions, although other laser wavelengths have been utilized with varying degrees of success. We present the case of a gentleman with a hypertrophic PWS who had an excellent response to Nd:YAG laser treatment. The increased tissue penetration of longer laser wavelengths may be of benefit to patients with hypertrophic PWS, and further research into this concept is warranted.

## 1. Introduction

Port-wine stain (PWS), also called nevus flammeus, is the most common congenital malformation of the capillaries and postcapillary venules, previously called “fire marks” or misnamed “flat hemangioma.” They occur in 0.1–2% of newborns without sex predilection but are less common in both Asians and African Americans [1]. At birth, the lesions are flat, but with age, they become hypertrophic and darker due to progressive vascular ectasia [2]. Histologic examination of these lesions reveals ectasia of the capillaries and postcapillary venules in the papillary and reticular dermis, with fibrosis and dilatation developing in later-stage lesions. They may occur anywhere on the body, but facial involvement is common and may be a significant source of psychological stress for the patient. It is often treated as a cosmetic condition, but depending on size and location, patients may show functional compromise of speaking, eating, or even vision. In approximately two-thirds of patients, vascular hyperplasia leads to the development of vascular nodules by the age of 50 [1]. Soft tissue hypertrophy usually occurs between 1 and 29 years of age, bony hypertrophy begins at an average age of 15 years, and nodules tend to develop in the age range of 15–53 years. Approximately 90% of PWS are located on the face in a trigeminal dermatome pattern. The majority of facial PWS (90%) are unilateral [3].

Interestingly, about 15–20% of children with PWS of the (V1) trigeminal dermatome are at risk for Sturge–Weber syndrome (SWS), a neurocutaneous disorder with vascular malformations in the cerebral cortex ipsilateral to PWS. Current literature suggests pathoanatomically redefining PWS as “a malformation resulting from differentiation-impaired endothelial cells with a progressive dilatation of immature venule-like vasculatures.” Recent discoveries have identified somatic mutations in the guanine nucleotide-binding protein, *G* alpha subunit *q* (GNAQ) (R183Q), phosphatidylinositol 3-kinase (PI3K), and activation of mitogen-activated protein kinase (MAPK), and PI3K pathway in skin lesions of PWS/SWS improved our understanding of the pathogenesis of these malformations [4].

Since the early 80s, vascular-specific lasers have been considered the gold standard in treating PWS, and classically pulsed dye lasers are regarded as the treatment of choice. However, the concept of selective photothermolysis with these devices was most applicable to patients with specific vascular phenotypes in superficial dermal layers. Therefore, some patients with hypertrophic port-wine stains are recalcitrant to pulse dye lasers. Research has suggested that over the last decade, meaningful treatment outcomes have not significantly improved, despite numerous technical innovations and pharmacological interventions [5]. These interventions have included new PDL systems with longer wavelengths (585 and 595 nm), epidermal cooling modalities, longer pulse durations, different light sources, and novel approaches altogether such as photodynamic therapy, pharmacological interventions, and combinations of the aforementioned treatments [6]. The importance of advances in patient outcomes for PWS is clear: patients are often stigmatized due to disfigurement, and this can have a serious impact on self-esteem and contribute to significant psychological stress [7].

## 2. Case Report

**Figure 1:**
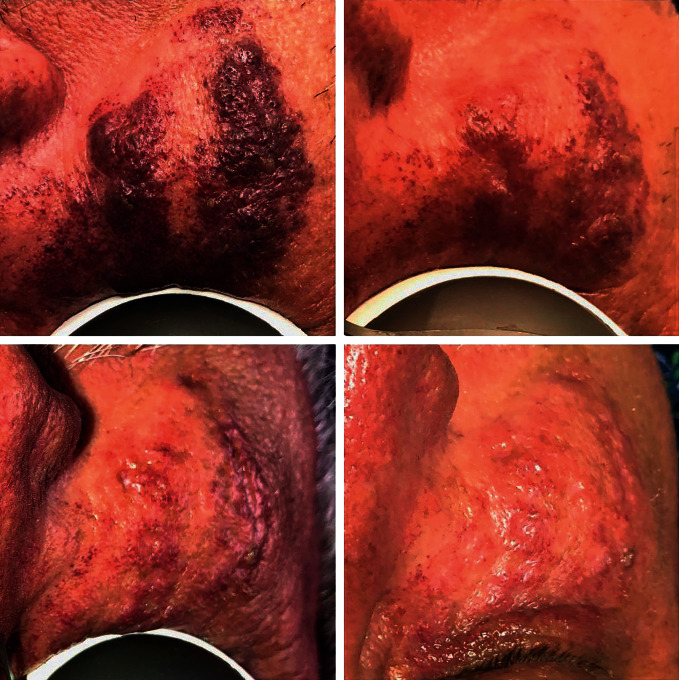
Port-wine stain after 3 Nd:YAG laser treatments.

A 60-year-old Caucasian male presented to our office for treatment of port-wine stain on his right cheek. The lesion had significant hypertrophy, and he had neither sought nor received prior treatment for condition. A pulsed dye laser was not available at our institution, so Nd:YAG laser was offered as an alternative treatment option with the patient's informed consent. Our previous experience with Nd:YAG therapy was for treatment of superficial vascular lesions, where it has an established track record in clinical practice [8]. After 3 treatments, the patient had dramatic improvement in appearance, with significant reduction in color and tissue hypertrophy (Figure 1).

## 3. Discussion

For treatment of port-wine stains, pulsed dye lasers have been regarded as the standard of care. This is primarily due to the excellent avidity of the 585 nm wavelength for oxyhemoglobin, and studies have shown the clinical benefit with low risk of side effects. However, the degree of lesion improvement and patient satisfaction vary significantly, and studies have shown that younger patients achieve better clearance with fewer treatments than older patients [9]. It has also been shown that patients with hypertrophic lesions experience higher complications and significantly less benefit from PDL therapy [10].

The 585 nm pulsed dye laser wavelength is excellent for treating superficial vascular lesions because of its high absorption coefficient for targeting oxyhemoglobin, but it has a significant shortcoming with regards to depth of tissue penetration. The 1064 nm wavelength may be less efficient against oxyhemoglobin as a target, but it has superior tissue penetration and a better ability to reach dermal vessels. This superior tissue penetration could potentially have a role in treating thicker, hypertrophied lesions such as the port-wine stains in older adult patients.

## 4. Conclusions

585 nm pulse dye lasers not only have an efficient wavelength for targeting oxyhemoglobin but also possess suboptimal tissue penetration for treatment of thicker lesions such as hypertrophic PWS. The 1064 nm Nd:YAG laser has better tissue penetration, but with decreased avidity for oxyhemoglobin. Instead of a one-size-fits-all approach to the treatment of PWS, it seems possible that patients may benefit from the use of different wavelengths depending on the degree of hypertrophy that is present in the vascular lesion. The optimal sequence of wavelengths is currently unclear, but we believe that a logical approach would be to use longer wavelengths initially, as this may decrease tissue hypertrophy and congestion, such that subsequent treatment with shorter wavelengths may be more successful.

## Data Availability

The data used to support the findings of this study are included within the article and linked in references.
